# Investigation of the reproducibility of the treatment efficacy of a commercial bio stimulant using metabolic profiling on flax

**DOI:** 10.1007/s11306-024-02192-1

**Published:** 2024-11-02

**Authors:** Kamar Hamade, Ophelie Fliniaux, Jean-Xavier Fontaine, Roland Molinié, Damien Herfurth, David Mathiron, Vivien Sarazin, Francois Mesnard

**Affiliations:** 1 UMRT INRAE 1158 BioEcoAgro, Laboratoire BIOPI, University of Picardie Jules Verne, Amiens, 80000 France; 2 Plateforme Analytique, University of Picardie Jules Verne, Amiens, 80000 France; 3 AgroStation, Rue de la Station, Aspach-le-Bas, 68700 France

**Keywords:** Flax, bio stimulants, metabolomics, systemic responses, lodging resistance

## Abstract

**Introduction and objectives:**

Since the use of a bio stimulant should provide a response to a problem that depends on the production system implemented (crops, plant model, soil, climate, the farmer’s practices…), the agricultural sector is facing concomitant challenges of choosing the best bio stimulant that suits their needs. Thus, understanding bio stimulant-plant interactions, at molecular level, using metabolomics approaches is a prerequisite, for the development of a bio stimulant, leading to an effective exploration and application of formulations in agriculture. AGRO-K®, is commercialized as a plant-based bio stimulant that improve vigor and enhance resistance to lodging in cereal crops. A recent previous untargeted metabolomics study has demonstrated the ability of this bio stimulant to improve wheat resistance to lodging, in real open-field conditions. However, the reproducibility of the impact of this bio stimulant in other filed crops is not yet investigated.

**Methods:**

Therefore, the present study aimed to assess the changes in primary and secondary metabolites in the roots, stems, and leaves of fiber flax (*Linum usitatissimum L*), treated with the bio stimulant, using NMR and LC-MS-based untargeted metabolomics approach.

**Results and conclusions:**

In addition to the previous result conducted in wheat, the present analysis seemed to show that this bio stimulant led to a similar pathway enhancement in flax. The pathways which seem to be reproducibly impacted are hydroxycinnamic acid amides (HCAAs), phenylpropanoids and flavonoids. Impacting these pathways enhance root growth and elongation and cell wall lignification, which can aid in preventing crop lodging. These results confirm that HCAAs, flavonoids, and phenylpropanoids could serve as signatory biomarkers of the impact of AGRO-K® on improving lodging resistance across various plant species.

## Introduction

The fact that the use of synthetic plant protection products is widely criticized and the need to increase productivity while improving plant growth in adverse environmental conditions, has recently brought plant bio stimulants into industry (du Jardin et al., 2020).

Bio stimulants are natural-based solutions that enhance plant growth and promote plant function to improve stress tolerance; they are assumed to have a lower impact on the environment or human health than chemical product (La Torre et al., 2016); (du Jardin et al., 2020). As studies with identical or similar bio stimulants have showed a different effectiveness data (Schütz et al., 2017), the choice of bio stimulant for achieving a specific objective is still a critical challenge. This is due, mainly, to the complexity of plant biochemical processes, to the variability in genotypic response, as well as to the pivotal modelling role exerted by both agronomic practices and environmental factors (Liliane et al., 2020). With this premise, we can point out that the empirical knowledge collected from different experimental conditions is of critical value for farmers (Li et al., 2022). Therefore, sufficient high-quality experimental data is required from the producers of Bio stimulants to support the claims they make on their products and provide valuable information and practical advice for the users (“Bio4safe database | Bio4safe,” 2021). Researches conducted on bio stimulants are more effective when they consider not only agronomic aspects such as crop yield, quality and ability to resist different types of stress, but they also include insights to understand the activity and the related mechanisms of action of the products.

A better understanding of the mode of action through which bio stimulants exert their impact can be achieved through metabolomics approaches which allow to determine the effects of bio stimulant in plants at the molecular level by the characterization of the set of metabolites present in plant tissue (Paul et al., 2019).

In our previous untargeted metabolomics work, we provided a strong evidence that metabolomic are helpful tool to evaluate the impact of bio stimulants on plants. In the study, we performed a metabolomic analysis to assess the impact of a commercial plant based bio stimulant containing galacturonic acid as elicitor, used in agriculture to reduce the risk of plant lodging. The results of the experiment demonstrate that, the treatment of wheat with this bio stimulant, enhance root growth and cell wall lignification, through the modulation of hydroxycinnamic acid amides (HCAAs), lignin and flavonoids biosynthetic pathways, in the different plant parts (Hamade et al., 2024). Thus, to ensure end-users’ confidence, understanding the mode of action of this biostimulant and the reproducibility of its effects on plant metabolism should be investigated through more quantitative assessments in field trials conducted on various plant models and under different cultivation conditions.

For this purpose, in the present work, the impact of this bio stimulant was investigated in two different fiber flax (*Linum usitatissimum L.*) cultures, using metabolomic analysis. The two cultures named C1 and C2 were conducted on the same soil, in the same time, but in different field, using different seed varieties.

## Materials and methods

### Agronomic field experiment

For the two cultures, the soil was sandy loam with a neutral pH (between 6.8 and 7.0). Between C1 and C2, the difference was the location, the seed variety and the harvest time point after the treatment with the bio stimulant. The culture C1, was conducted in Mars in La Poterie Cap d’Antifer (France), using Daurea linseeds, and the culture C2 located 10 Km from C1, was conducted in Mars in Saint-Jouin-Bruneval (France), using Melina linseeds. The bio stimulant AGRO-K^®^ was provided by Nufarm (Colombes, France). For the two cultures, plants were treated with the bio stimulant according to the manufacturer’s recommendations (https://nufarm.com): foliar spraying, 1 month after seed sowing, at a dose of 1 kg/ha for C1 and 1 kg/ha or 2 kg/ha, for C2. The experimental set-up was a completely randomized block design with one untreated control block for each of C1 and C2, one AGRO-K^®^ treated block for C1 and two AGRO-K^®^^®^ treated blocks for C2. Control and treated plants were harvested after 10 and 19 days of bio stimulant application (D10 and D19) for C1, and after 12 and 21 Days (D12 and D21), for C2. During harvest, the roots from each plant were washed with running water to remove all remaining soil and the plants were immediately frozen in liquid nitrogen. Samples were stored for 48 h at -80 °C and then freeze-dried. The dried leaves, stems, and roots of each plant were separated and ground into a fine powder using a MM200 ball mill (Retsch Gmbh & Co, Germany). Eight randomly independent plants were chosen, for each condition and time point (*n* = 8).

## Metabolite extraction

Metabolites extraction protocol was done following the same protocol adapted in our previous study (Hamade et al., 2024). 100 mg of powdered leaves, stems, and roots, were mixed with 800 µL of water/methanol (1:1). The mixture was placed in a ThermoMixer^®^ (Eppendorf AG, Hamburg, Germany), for 10 min, at 60 °C and 6.708 ×g, sonicated for 30 min at 60 °C using an ultrasonic bath at 35 kHz, and then centrifuged at 4 °C for 10 min at 18,845 ×g. 400 µL of the clear supernatant was transferred into a 2 mL Eppendorf tube and the remaining residue was extracted a second time by adding 400 µL of water/methanol (1:1) and the mixture was placed again in a Thermomixer^®^, sonicated and then centrifuged following the above-described procedure. 400 µL of the clear supernatant from this extraction was added to the 400 µL previously transferred to the Eppendorf tube. A third extraction was done by adding another 400 µL of water/methanol (1:1) to the remaining residue and repeating the steps of agitation, sonication, and centrifugation. The only parameter that varied during the different extractions was the sonication time (20 min for the second extraction and 10 min for the third extraction). A final volume of 1.2 mL of extract was obtained, of which 800 µL was taken for NMR analysis while the remaining 400 µL was subsequently used for LC-MS analysis.

## Metabolite analysis by NMR

Samples prepared for NMR analysis were adjusted to a pH of 6.00 ± 0.02, dried in a vacuum concentrator, and then redissolved in 800 µL of a deuterated solvent prepared in a mixture of (1:1) methanol-d4:KH_2_PO_4_ buffer (0.1 M) in D_2_O containing 0.0125% TMSP as a standard for calibration and NaN_3_ (0.6 mg/mL), to prevent bacterial contamination. The mixture was vortexed at 60 °C for 10 min using thermomixer, sonicated for 5 min and then centrifuged at 4 °C for 10 min, and the supernatant was transferred into 5 mm NMR tubes.

The NMR experiments for untargeted metabolomic profiling was performed according to our previously described method (Hamade et al., 2024), using a Bruker Avance III 600 MHz spectrometer, equipped with a 5 mm multinuclear detection broadband, and z-gradient (TXI 5 mm tube). The NOESY-1D water suppression pulse sequence was used and generated spectra were collected at 131 K data points using 256 scans, and a spectral width of 8417 Hz with a relaxation delay of 25 s. The ^1^H-NMR spectra were automatically phase, baseline corrected and referenced to the TMSP signal (δ = 0 ppm), using the software Topspin (version 3.5) from Bruker (Deborde et al., 2019). 1D NOESY spectra were exported to ASCII file format, using TopSpin 3.2 software and the data from each plant part (leaves, stems, and roots) were imported separately into MATLAB software (version 2017b, Mathworks Inc, Natick, MA, USA), where the baseline was corrected with the package airPLS 2.0 (Gan et al., 2006). All spectra were aligned simultaneously using the icoshift algorithm (v 1.2.1) with manually defined alignment bins. Then, with the use of the dynamic adaptive binning (DAB) algorithm (Anderson et al., 2011), specific integration intervals of the spectra called “bins” were defined. The data set obtained for each observation (sample) consisting of a few hundred variables (bins) was used for statistical analysis. 2D ^1^H JRES NMR spectra were acquired using 16 scans per 64 increments that were accumulated into 64 K data points, using spectral widths of 8417.5 Hz in the F2 dimension and 50 Hz in the F1 dimension. The 2D HSQC spectra were acquired using 64 scans per 256 increments that were collected into 4 K data points, with spectral widths of 8417.5 Hz in F2 and 50 Hz in F1.

## Metabolite analysis by LC-MS/MS

Extracts of leaves, stems, and roots of flax were diluted 50, 30, and 5 times, respectively, with methanol/water (50/50). Samples were filtered through PTFE syringe filters with pore size of 0.22 μm and placed in vials for further LC-MS analysis. A quality control (QC) sample, prepared by pooling 10 µL extract from each test sample, was analyzed multiple times throughout the chromatographic sequence, to assess the stability and reproducibility of the analysis. Untargeted LC-MS analysis was performed using an ACQUITY UPLC I-class chain coupled to a Vion IMS Q-TOF high resolution mass spectrometer, equipped with an electrospray (ESI) (Waters, Manchester, UK) ionization source (Z-spray) and an additional spray for the reference compound (Lock Spray).

In brief, liquid chromatography was performed using a Kinetex C18 column (1.7 μm, 100 mm x 2.1 mm, Phenomenex, Torrance, CA, USA), with H2O as phase A and methanol as phase B, both with 0.01% formic acid. The column temperature was maintained at 50 °C. The total run time was 11 min and the flow rate was 0.5 mL/min and the gradient was: 0 min, 5% B; 7 min, 95% B; 9 min, 5% B and was held for 2 min. The injection volume was 1 µL. ESI parameters are: Cone voltage, 120 V; source offset, 20 V; source temperature, 120 °C; desolvation gas temperature, 450 °C; desolvation gas flow, 800 L/h; and cone gas flow, 50 L/h, in positive or negative modes. Time-of-flight (TOF) MS was operated in sensitive mode. The data were acquired with a mass range of m/z 50–2000 Da. For MS/MS experiments, argon was used as collision gas and the collision energy value (see Table 1), was optimized to reach a relative intensity between 10 and 20% for each selected precursor ion. The software UNIFI Waters Scientific Information System (v 1.9.4) was used for the processing of spectral data and for the generation of untargeted metabolomics data matrix, including retention time and m/z, sample names, and ion intensities for each sample set. Further data cleaning was performed to remove metabolic features present in the blank and those with relative standard deviation (% RSD) greater than 35% in the quality control (QC).

## Statistical analysis

The obtained datasets for NMR and LC-MS for each of the three plant parts of flax were subjected separately to a multivariate non-targeted analysis, using Matlab software (The MathWorks, Natick, USA). A multi-block orthogonal partial least squares discriminant analysis (Multiblock OPLS-DA) were generated, in order to study the classification of the different analyzed groups (Boccard & Rutledge, 2013). For both C1 and C2, two classes were applied for the construction of the OPLS-DA framework: control and treated samples. This classification was designed to summarize the discriminant information between control and treated samples based on the predictive component(s). A permutation test was conducted to assess the OPLS-DA model validity.

An analysis of the OPLS-DA loading plots was then conducted to identify the metabolites found to be most relevant to the discrimination between groups. The Wilcoxon Rank Sum test from R’s base statistics package was used to evaluate significant differences in metabolite content between the analyzed groups, with significance levels set at *p* < 0.05, *p* < 0.01, and *p* < 0.001.

## Results

**Fig. 1:**
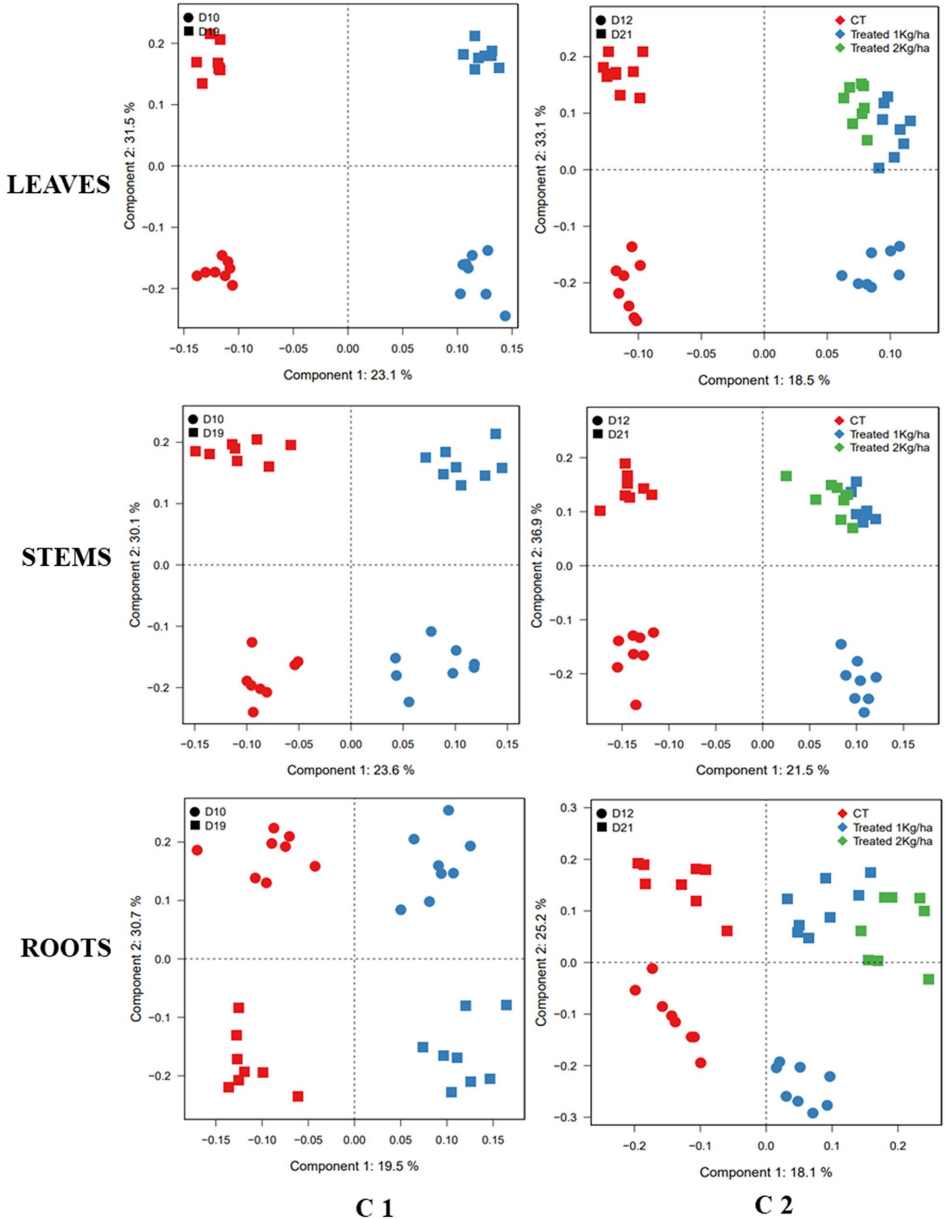
Score plot of multi-block orthogonal partial least squares discriminant analysis (OPLS-DA) of metabolite content in Flax leaves, stems, and roots, based on ^1^H-NMR and LC-MS data (Pos/Neg ion mode), for control (Red) and treated (Blue and green) samples, at different time points: D10, D19 for C1 and D12 and D21 for C2

The score plot of OPLS-DA, shown in Fig. 1, indicates that for the two cultures, C1 and C2, the biostimulant-treated samples were separated from the control samples, based on the predictive score plot (component 1), across various plant parts. These observations highlighted the differential metabolic profiles, in leaves, stems and roots, between the bio stimulant treated and control samples, meaning that flax plants were responding to the bio stimulant treatment.

Additionally, the OPLS-DA showed that for both control and treated samples, at D10 and D19 for C1, and at D12 and D21 for C2, the samples were separated according to the orthogonal score plot (component 2). These results provide relevant additional information about the metabolic variability related to the developmental stage of flax.

**Fig. 2:**
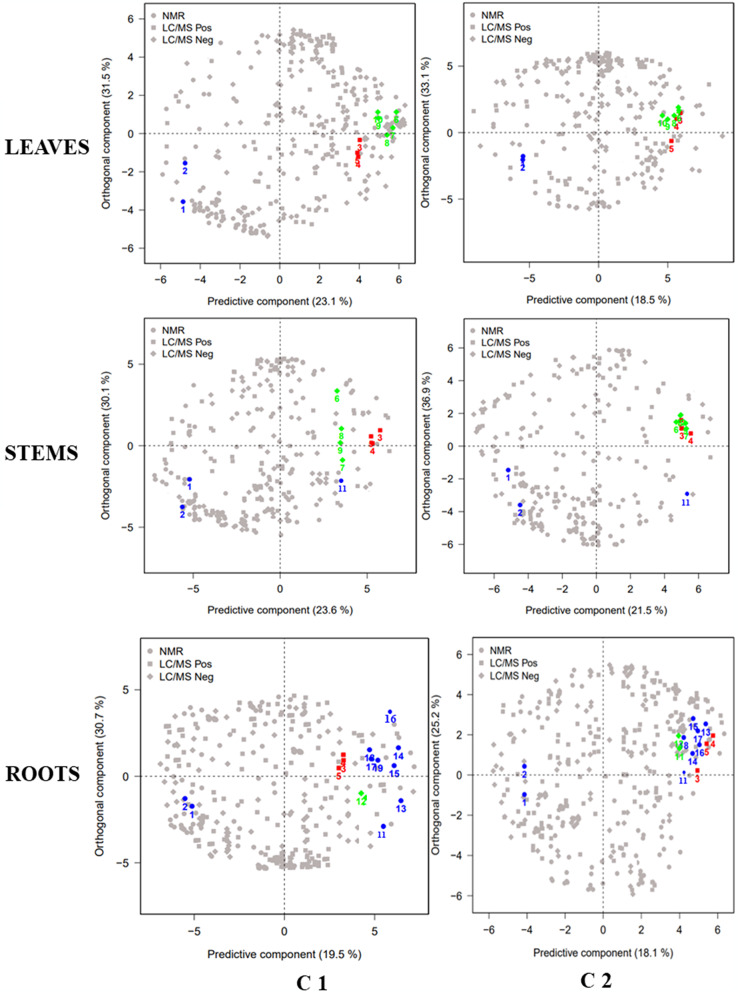
OPLS-DA loading plot, showing the distribution of metabolites responsible for the discrimination of the corresponding score plot (Fig. 1), of Flax leaves, stems and roots, in control and treated conditions, at different time-points: D10, D19 for C1 and D12, D21 for C2. • discriminant variables detected with NMR; ▪ detected with LC-MS in positive ionization; ⬪ detected with LC-MS in negative ionization. (1)Arginine; (2)Phenylalanine; (3)N-feruloyl-putrescine; (4)N-feruloyl-spermine; (5)N-coumaroyl-feruloyl-spermine; (6)Vitexin; (7)Triticuside-A; (8)Schaftoside; (9)Vicenin-2; (10)Lucenin-2; (11)Coniferyl Alcohol; (12) Guaiacyl (8-O-4)Syringyl (8–8) Guaiacyl; (13)Aspartate; (14)Glycine; (15)Proline; (16)Glutamate; (17)Succinic Acid; (18)Fumaric Acid: (19)Malic Acid

For the C2 culture, the effect of the bio stimulant was investigated at two dose treatment (1 kg/ha and 2 kg/ha), at D21 after treatment. The OPLS-DA analysis revealed no distinct groupings between samples treated with 1 kg/ha and those treated with 2 kg/ha, in different plant part. In addition, a Wilcoxon Rank Sum test was performed on the datasets for control and treated samples, to verify whether there was a significant difference between the two doses of AGRO-K^®^ application. The test also highlighted no significant differences in metabolite content of treated samples after 21 Day (Data not shown). Thus, samples treated with different dose of bio stimulant could be considered as a same group. These observation provides information necessary for farmers to select 1 kg/ha as an effective dose to get a response to the bio stimulant, in flax culture with lower cost. The corresponding loading plots of the OPLS-DA obtained from NMR and LC-MS data represented in Fig. 2, showed the distribution of metabolites in control and treated samples in the different plant part, for the two cultures C1 and C2.

The next step to compare control and treated samples consisted of searching the discriminant metabolites in each plant part, in order to identify the metabolic pathways affected by the bio stimulant treatment.

The identification of discriminant metabolites detected in NMR, was based on the matching of the 1D and 2D NMR spectra with the spectra of standard reference, available in the database developed in our laboratory.

For the discriminant metabolites detected in LC-MS, some features were identified by matching the exact masses, retention times and MS/MS spectrum to those of an analytical reference standard. Others were annotated with accurate masses and MS/MS spectrum of molecules available in an accessible mass spectral library. The identification was also confirmed using previous publications on the same molecules (Tchoumtchoua et al., 2019) (Elboutachfaiti et al., 2022) (Chantreau et al., 2014) (Morreel et al., 2010) (Tais et al., 2021) (Hanhineva et al., 2012) (Moheb et al., 2011) (Li et al., 2018). All information about identification of the discriminant metabolites are indicated in Table 1.

**Table 1:**
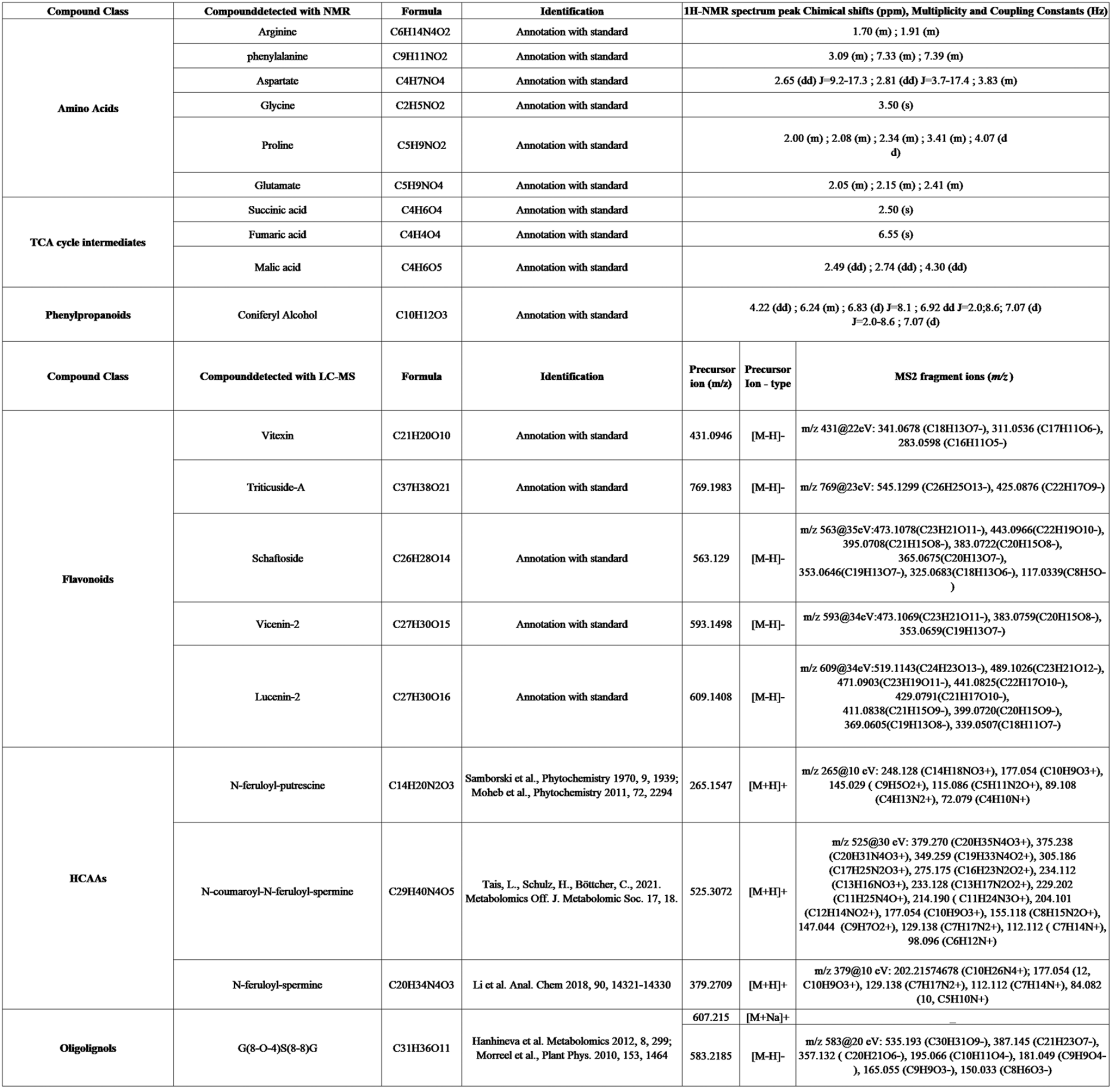
List of discriminant metabolites identified with ^1^H-NMR and LC-MS

**Fig. 3:**
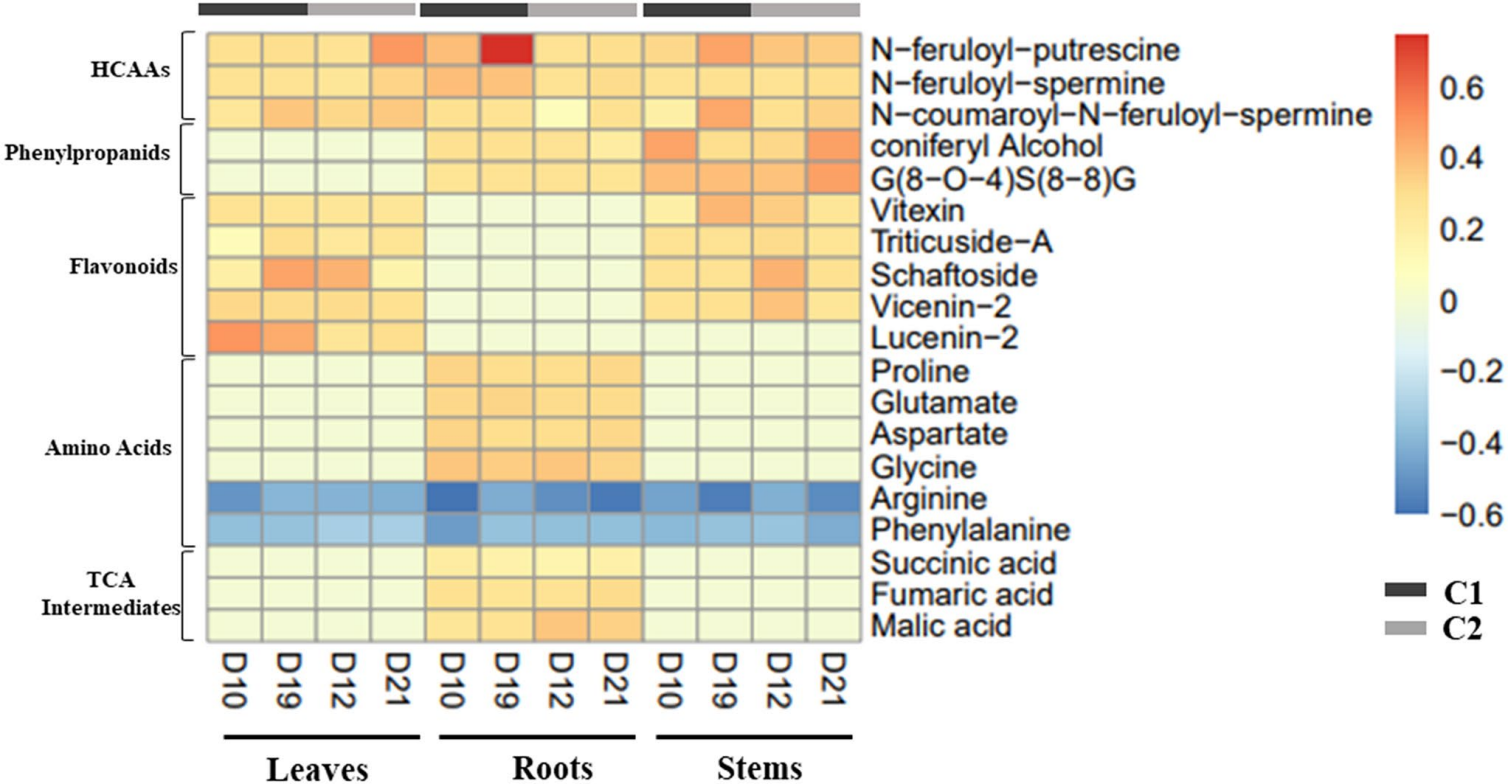
Comparison of major metabolite content reorganization in the Leaves, stems and roots, of Flax, after treatment with the bio stimulant at different time points: D10, D19 for C1 and D12, D21, for C2. Each color represents a specific value of the mean relative response ratio T/C (content in plant treated by the bio stimulant/content in control plant), on a logarithmic scale (log10). Positive values represent a higher content and Negative values represent a lower content of metabolites in treated plants compared to those in the control. Changes in metabolite content were considered statistically significant when p-values were lower than 0.05, after the Wilcoxon Rank Sum test

Fig. 3 provides a heat map with the relative fold changes (in log 10) of the metabolites that contribute the most to the discrimination of the control and treated samples, in the corresponding score plot OPLS-DA of the different plant parts, for the two culture.

In the roots, stems and leaves, of the two culture, a significant increase in the amount of hydroxycinnamic acid amides (HCAAs), was observed, in the treated samples compared the control samples, at different harvest time points. For both cultures, the largest significant fold increase in HCAAs content was observed in N-feruloyl putrescine, with a highest T/C ratios (calculated by the formula: ratio T/C = content plant treated with AGRO-K^®^ /content control plant) of 5.67 in the roots of C1 culture at D19 and 3.11 in the leaves of C2 culture, at D21. An accumulation in the similar manner was also observed for N-feruloyl-spermine and N-coumaroyl-N-feruloyl-spermine, in the different plant parts.

Furthermore, a significant decrease in the level of an amino acid arginine was observed in the stems, leaves and roots of flax, after the treatment with the bio stimulant, with a T/C ratio ≤ 0.4, over the harvest period, in the two culture C1 and C2. This amino acid is a direct precursor for the synthesis of polyamines such as putrescine, spermidine, and spermine, through the action of arginine decarboxylase (ADC), spermidine synthase, and spermine synthase (Gill & Tuteja, 2010). HCAAs result from the conjugation of polyamines with hydroxycinnamic acids, such as p-coumaric, ferulic, and caffeic acids, together with some glycosylated forms (Liu et al., 2022). Therefore, the observed accumulation in the content of N-feruloyl-putrescine, N-feruloyl-spermine and N-coumaroyl-N-feruloyl-spermine, in the treated samples provides an explanation for the simultaneous bio stimulant-induced decrease in arginine level, which can be converted into putrescine and spermine, essential for production of the above mentioned HCAAs.

A significant increase in the content of phenylpropanoids such as coniferyl alcohol and oligolignol such as guaiacyl (8-O-4)syringyl (8–8) guaiacyl (G(8-O-4)S(8–8)G), was observed in the flax stems and roots, after the application of the bio stimulant. For coniferyl alcohol, the largest increase was observed in the stems, when compared to the roots, with a T/C ratios of 2.91 and 2.03, for C1 culture, at Day 10 and Day 19 respectively and 2.09 and 2.97 for C2 culture, at Day 12 and Day 21, respectively. The increase in the amount of G(8-O-4)S(8–8)G followed the same trend, with a T/C ratios of 2.42 and 2.95, in the stems of C1 culture, at Day 10 and Day 19 respectively and 2.09 and 2.97 in the stems of C2 culture, at Day 12 and Day 21, respectively.

In addition, in the two cultures, an increase in the content of flavonoids such as triticuside-A, vitexin, schaftoside, vicenin-2 and lucenin-2, was observed in the stems and leaves of treated samples compared to those in the control.

Concurrently, with the accumulation of HCAAs, phenylpropanoids, and flavonoids, the application of the bio stimulant appeared to decrease the amount of phenylalanine, in the stems, roots and leaves of the two flax cultures. In fact, phenylalanine ammonia lyase (PAL), is a key gateway enzyme that links the primary and secondary metabolism, mainly through the phenylpropanoid pathway, which branches into a network of other pathways. This enzyme catalyzes the deamination of phenylalanine giving rise to cinnamic acid. This latter is then serves as precursor leading to the generation of various phenolic compounds such as HCAAs, flavonoids and lignins (Kong, 2015). Thus, the decrease in the amount of phenylalanine observed in the treated flax samples, could be explained by the redirection of the plant to induce deamination of this compound in order to yield more cinnamic acid, which is involved in the biosynthesis of HCAAs, phenylpropanoids, and flavonoids.

In summary, the quantitative pathway analysis of the differentially abundant metabolites in roots, stems and leaves of flax, a dicotyledonous plant, postulated that the application of AGRO-K^®^, induce a changes in the metabolic pathways of HCAAs, flavonoids and phenylpropanoids. A similar postulated framework, describing AGRO-K^®^-induced events was observed in our previous metabolomic study, conducted in wheat, a monocotyledonous plant. Studies in several plant species have shown a positive correlation between HCAAs content and root growth rate and elongation (BIONDI et al., 1993) (Tang & Newton, 2005) (Couée et al., 2004) (Tarenghi & Martin-Tanguy, 1995) (Hummel et al., 2002) (Ebeed et al., 2017). In addition, HCAAs, were found to increase cell wall thickness (Zeiss et al., 2021) (Gunnaiah et al., 2012) (Graça, 2010); Macoy et al., 2022) (Macoy et al., 2015).

Several studies have shown that the lignin content in stems and roots of plants was significantly related to lodging resistance (Li et al., 2021). Lignin is mainly present in secondary-thickened cell walls, provide structural support and giving rigidity and strength to stems to stand upright (Yi Chou et al., 2018).

Flavonoids also has long been known as a key regulator of physiological processes, including root gravitropism and branching (Buer et al., 2006); (Buer & Muday, 2004); (Brown et al., 2001); (Buer & Djordjevic, 2009) (Peer et al., 2004).

Based on the abovementioned, it may be assumed that AGRO-K^®^, led to a similar pathway enhancement and with common discriminant metabolites, in different plant species. The pathways which seem to be reproducibly impacted are associated with root development, soil anchorage, and cell wall stiffening and lignification. The findings herein, therefore, contribute towards the generation of a fundamental knowledgebase describing the molecular mechanisms underlying the effects of this bio stimulant on enhancing plant vigor and improving resistance to lodging and provide actionable knowledge necessary for industries and farmers to confidently using this bio stimulant, into agronomic practices for different agriculture.

Moreover, a specific metabolic change was observed only in Flax (not in wheat), and especially in the roots, after the treatment with the bio stimulant. This was the case of the amino acid and the tricarboxylic acid (TCA) intermediates profile. The general effect of the bio stimulant was the significant increase in the concentration of amino acids such as proline, glutamate, aspartate, glycine and of tricarboxylic acid (TCA) such a succinic, fumaric and malic acid, only in roots of flax. A study conducted by Biancucci et al., showed that proline can promote root elongation in Arabidopsis plant via the modulation of the size of root meristematic zone likely by controlling cell division and, in turn, by modulating the ratio between cell division and cell differentiation (Biancucci et al., 2015). Recent studies have identified that glutamate usually exerts signaling role by its receptors (GLRs), which are also capable of binding to other amino acids. These receptors, when activated by amino acids, can trigger a series of physiological processes such as the stimulation of lateral roots growth and development (Kong et al., 2015). Another study showed that, in *Brassica napus*, aspartate induce cell division and promote root development and elongation, through the modulation of the ethylene and/or ethylene signaling pathway (Lemaire et al., 2013). In Habanero Pepper (*Capsicum chinense*), glycine promotes root hair growth that allows access to a greater surface area and volume of soil, which can favor both the acquisition of water and nutrients (Domínguez-May et al., 2013).

Furthermore, amino acids generally play a several roles in plants, from serving as basic building blocks of proteins to being a signal metabolites that interact with numerous metabolic pathways which stimulate plant growth (Pratelli & Pilot, 2014). Following degradation, amino acids’ carbon skeletons are generally converted into precursor intermediates of the tricarboxylic acid (TCA) cycle that produce energy in the form of ATP (Hildebrandt et al., 2015).This produced energy can fuel a wide-range of energy-demanding biochemical processes that contribute in plant growth and development such as gene expression, mobility and metabolism (De Col et al., 2017).

Herein, the simultaneous increase in the amount of amino acids and tricarboxylic acid intermediates in the roots of flax treated with the bio stimulant, led to the hypothesis, that treatment with bio stimulant induce ATP production, through the amino acid accumulation, in order to enhance root growth and elongation.

**Fig. 4:**
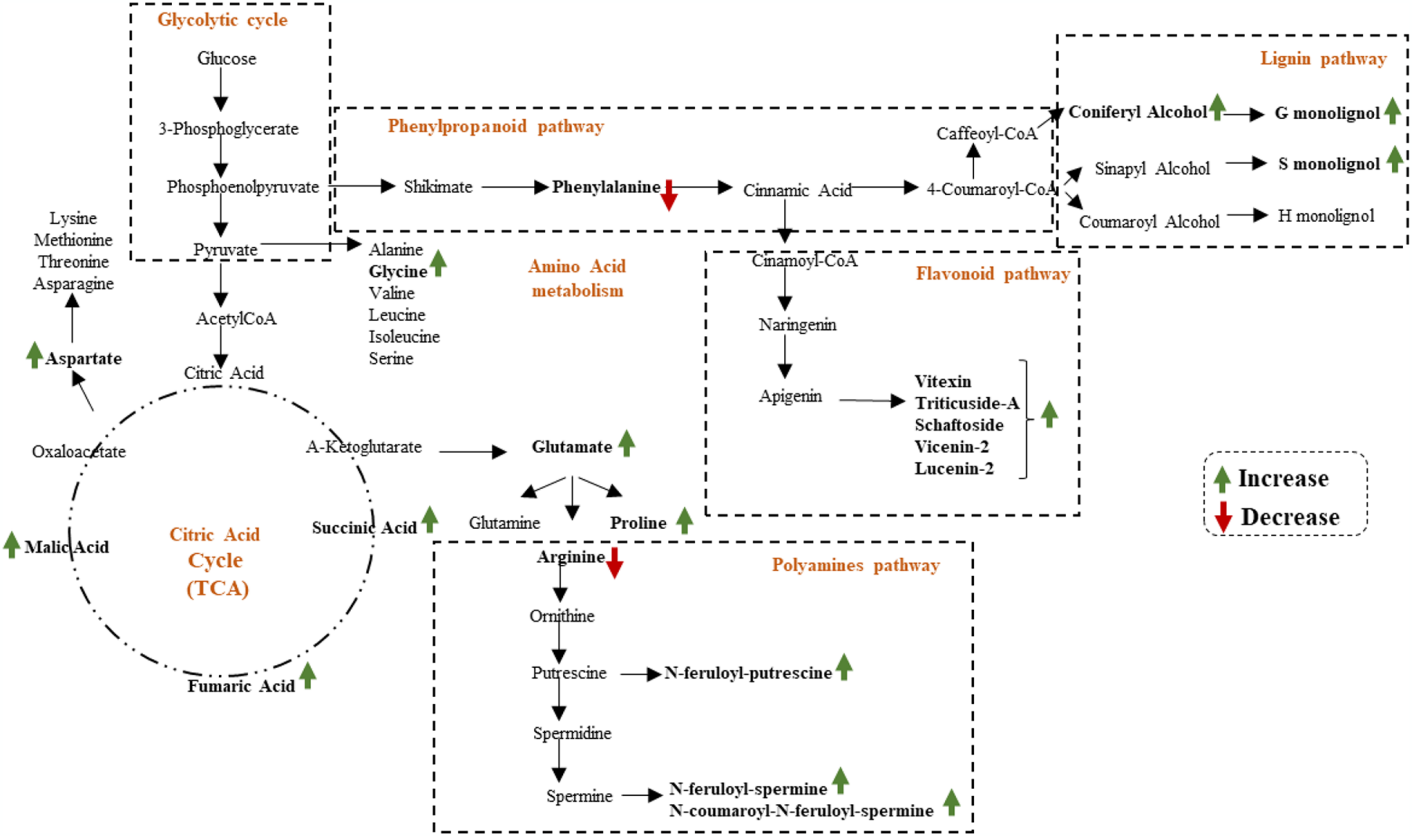
Major changes in the primary and secondary metabolic pathways, of the two culture of flax, after treatment with the bio stimulant. The proposed metabolic pathways were based on the literature. The green up-arrow and red down-arrow indicate higher and lower levels of metabolites, respectively, in bio stimulant treated, compared with the control flax

Major changes in the primary and secondary metabolic pathways of roots, stems and leaves of flax after AGRO-K^®^ treatment, as well as the proposed relations between these metabolic pathways are presented in balance sheet shown in Fig. 4.

Agro-K^®^ is composed of a mixture of potassium oxide (50%), phosphorus oxide (32%), amino acids (10%), as well as galacturonic acid (5%) extracted from the Nopal cactus. Galacturonic acid is regarded as an efficient elicitor used for activating plant defense responses (Wan et al., 2021). This compound has been reported as Damage Associated Molecular Pattern (DAMP), that can be recognized by Pattern Recognition Receptor (PRRs), responsible for the stimulation of the immune system (Ferrari et al., 2013). Several studies on various plant models showed that treatment with galacturonic acid, as elicitor, accumulates transcripts such as PAL, which constitutes the point of connection between the primary metabolism of shikimate, leading to aromatic amino acids and the secondary metabolism of phenylpropanoids leading to flavonoids and lignin production, and stimulates changes in defense genes, including the salicylic acid, which is known to be involved in strengthening the cell wall by increasing polyamine, lignin and callose biosynthesis (Butselaar and Ackerveken, 2020) (Ochoa-Meza et al., 2021) (Canales et al., 2019) Thus, the mechanism of action of the bio stimulant could be attributed to the elicitation effect of galacturonic acid.

## Conclusion

The identification of various foliar biomarkers linked with response to elicitation suggests that its quantifications in controlled or field trials could be a huge time and cost saver for screening new plant defense stimulators or extending the use of stimulators based over Damage Associated Molecular Pattern.

For example, in our case, a direct quantification of flavonoids (Gitelson et al., 2017), and HCAAs (Shim & Jeong, 2019), in field conditions, using the non-destructive near-infrared approaches could be proposed, in order to reduce time and cost required for the assessment of the bio stimulant performance.

## Data Availability

No datasets were generated or analysed during the current study.

## Sample availability

Samples of the compounds are available on request from the corresponding author.

## References

[CR1] Anderson, P., Mahle, D., Doom, T., Reo, N., Delraso, N., & Raymer, M. (2011). Dynamic adaptive binning: An improved quantification technique for NMR spectroscopic data. *Metabolomics*, *7*, 179–190. 10.1007/s11306-010-0242-7

[CR2] Biancucci, M., Mattioli, R., Moubayidin, L., Sabatini, S., Costantino, P., & Trovato, M. (2015). Proline affects the size of the root meristematic zone in Arabidopsis. *Bmc Plant Biology*, *15*, 263. 10.1186/s12870-015-0637-8 26514776 10.1186/s12870-015-0637-8 PMC4625561

[CR3] Bio4safe database | Bio4safe [WWW Document], n.d. URL https://bio4safe.eu/ (accessed 2.9.24).

[CR4] Biondi, S., Mengoli, M., Mott, D., & Bagni, N. (1993). Hairy root cultures of Hyoscyamus muticus: effect of polyamine biosynthesis inhibitors. *Hairy Root Cult Hyoscyamus Muticus Eff Polyam Biosynth Inhib*, *31*, 51–58.

[CR5] Boccard, J., & Rutledge, D. N. (2013). A consensus orthogonal partial least squares discriminant analysis (OPLS-DA) strategy for multiblock Omics data fusion. *Analytica Chimica Acta*, *769*, 30–39. 10.1016/j.aca.2013.01.022 23498118 10.1016/j.aca.2013.01.022

[CR6] Brown, D. E., Rashotte, A. M., Murphy, A. S., Normanly, J., Tague, B. W., Peer, W. A., Taiz, L., & Muday, G. K. (2001). Flavonoids act as negative regulators of auxin transport in vivo in arabidopsis. *Plant Physiology*, *126*, 524–535. 10.1104/pp.126.2.524 11402184 10.1104/pp.126.2.524 PMC111146

[CR7] Buer, C. S., & Djordjevic, M. A. (2009). Architectural phenotypes in the transparent testa mutants of Arabidopsis thaliana. *Journal Of Experimental Botany*, *60*, 751–763. 10.1093/jxb/ern323 19129166 10.1093/jxb/ern323 PMC2652062

[CR8] Buer, C. S., & Muday, G. K. (2004). The transparent testa4 Mutation Prevents Flavonoid Synthesis and Alters Auxin Transport and the Response of Arabidopsis Roots to Gravity and Light. *The Plant Cell*, *16*, 1191–1205. 10.1105/tpc.020313 15100399 10.1105/tpc.020313 PMC423209

[CR9] Buer, C. S., Sukumar, P., & Muday, G. K. (2006). Ethylene modulates flavonoid accumulation and gravitropic responses in roots of Arabidopsis. *Plant Physiology*, *140*, 1384–1396. 10.1104/pp.105.075671 16489132 10.1104/pp.105.075671 PMC1435817

[CR11] Canales, F. J., Montilla-Bascón, G., Rispail, N., & Prats, E. (2019). Salicylic acid regulates polyamine biosynthesis during drought responses in oat. *Plant Signaling & Behavior*, *14*, e1651183. 10.1080/15592324.2019.1651183 31382811 10.1080/15592324.2019.1651183 PMC6768256

[CR12] Chantreau, M., Portelette, A., Dauwe, R., Kiyoto, S., Crônier, D., Morreel, K., Arribat, S., Neutelings, G., Chabi, M., Boerjan, W., Yoshinaga, A., Mesnard, F., Grec, S., Chabbert, B., & Hawkins, S. (2014). Ectopic Lignification in the Flax lignified bast fiber1 Mutant Stem Is Associated with Tissue-Specific Modifications in Gene Expression and Cell Wall Composition[C][W]. *The Plant Cell*, *26*, 4462–4482. 10.1105/tpc.114.130443 25381351 10.1105/tpc.114.130443 PMC4277216

[CR13] Couée, I., Hummel, I., Sulmon, C., Gouesbet, G., & El Amrani, A. (2004). Involvement of polyamines in root development. *Plant Cell Tissue Organ Cult*, *76*, 1–10. 10.1023/A:1025895731017

[CR14] De Col, V., Fuchs, P., Nietzel, T., Elsässer, M., Voon, C. P., Candeo, A., Seeliger, I., Fricker, M. D., Grefen, C., Møller, I. M., Bassi, A., Lim, B. L., Zancani, M., Meyer, A. J., Costa, A., Wagner, S., & Schwarzländer, M. (2017). ATP sensing in living plant cells reveals tissue gradients and stress dynamics of energy physiology. *eLife*, *6*, e26770. 10.7554/eLife.26770 28716182 10.7554/eLife.26770 PMC5515573

[CR15] Deborde, C., Fontaine, J. X., Jacob, D., Botana, A., Nicaise, V., Richard-Forget, F., Lecomte, S., Decourtil, C., Hamade, K., Mesnard, F., Moing, A., & Molinié, R. (2019). Optimizing 1D 1H-NMR profiling of plant samples for high throughput analysis: extract preparation, standardization, automation and spectra processing. *Metabolomics Off J Metabolomic Soc*, *15*, 28. 10.1007/s11306-019-1488-3 10.1007/s11306-019-1488-3 PMC6394467 30830443

[CR16] Domínguez-May, Á. V., Carrillo-Pech, M., Barredo-Pool, F. A., Martínez-Estévez, M., Us-Camas, R. Y., Moreno-Valenzuela, O. A., & Echevarría-Machado, I. (2013). A Novel Effect for Glycine on Root System Growth of Habanero Pepper. *Journal Of The American Society For Horticultural Science*, *138*, 433–442. 10.21273/JASHS.138.6.433

[CR17] du Jardin, P., Xu, L., & Geelen, D. (2020). Agricultural Functions and Action Mechanisms of Plant Biostimulants (PBs). *The Chemical Biology of Plant Biostimulants* (pp. 1–30). Wiley. 10.1002/9781119357254.ch1

[CR18] Ebeed, H. T., Hassan, N. M., & Aljarani, A. M. (2017). Exogenous applications of Polyamines modulate drought responses in wheat through osmolytes accumulation, increasing free polyamine levels and regulation of polyamine biosynthetic genes. *Plant Physiol Biochem PPB*, *118*, 438–448. 10.1016/j.plaphy.2017.07.014 28743037 10.1016/j.plaphy.2017.07.014

[CR19] Elboutachfaiti, R., Molinié, R., Mathiron, D., Maillot, Y., Fontaine, J. X., Pilard, S., Quéro, A., Brasselet, C., Dols-Lafargue, M., Delattre, C., & Petit, E. (2022). Secondary Metabolism Rearrangements in Linum usitatissimum L. after Biostimulation of Roots with COS Oligosaccharides from Fungal Cell Wall. *Molecules*, *27*, 2372. 10.3390/molecules27072372 35408773 10.3390/molecules27072372 PMC9000297

[CR20] Ferrari, S., Savatin, D. V., Sicilia, F., Gramegna, G., Cervone, F., & Lorenzo, G. D. (2013). Oligogalacturonides: plant damage-associated molecular patterns and regulators of growth and development. Front. *Plant Science*, *4*, 49. 10.3389/fpls.2013.00049 10.3389/fpls.2013.00049 PMC3595604 23493833

[CR21] Gan, F., Ruan, G., & Mo, J. (2006). Baseline correction by improved iterative polynomial fitting with automatic threshold. *Chemom Intell Lab Syst*, *82*, 59–65. 10.1016/j.chemolab.2005.08.009. Selected Papers from the International Conference on Chemometrics and Bioinformatics in Asia.

[CR22] Gill, S., & Tuteja, N. (2010). Polyamines and abiotic stress tolerance in plants. *Plant Signaling & Behavior*, *5*, 26–33. 10.4161/psb.5.1.10291 20592804 10.4161/psb.5.1.10291 PMC2835953

[CR23] Gitelson, A., Chivkunova, O., Zhigalova, T., & Solovchenko, A. (2017). In situ optical properties of foliar flavonoids: Implication for non-destructive estimation of flavonoid content. *Journal Of Plant Physiology*, *218*, 258–264. 10.1016/j.jplph.2017.08.009 28915504 10.1016/j.jplph.2017.08.009

[CR24] Graça, J. (2010). Hydroxycinnamates in suberin formation. *Phytochemistry Reviews*, *9*, 85–91. 10.1007/s11101-009-9138-4

[CR25] Gunnaiah, R., Kushalappa, A. C., Duggavathi, R., Fox, S., & Somers, D. J. (2012). Integrated Metabolo-Proteomic Approach to Decipher the Mechanisms by Which Wheat QTL (Fhb1) Contributes to Resistance against Fusarium graminearum. *PLOS ONE*, *7*, e40695. 10.1371/journal.pone.0040695 22866179 10.1371/journal.pone.0040695 PMC3398977

[CR26] Hamade, K., Fliniaux, O., Fontaine, J. X., Molinié, R., Petit, L., Mathiron, D., Sarazin, V., & Mesnard, F. (2024). NMR and LC–MS-based metabolomics to investigate the efficacy of a commercial bio stimulant for the treatment of wheat (Triticum aestivum). *Metabolomics*, *20*, 58. 10.1007/s11306-024-02131-0 38773056 10.1007/s11306-024-02131-0 PMC11108958

[CR27] Hanhineva, K., Rogachev, I., Aura, A. M., Aharoni, A., Poutanen, K., & Mykkanen, H. (2012). Identification of novel lignans in the whole grain rye bran by non-targeted LC-MS metabolite profiling. *Metabolomics*, *8*, 399–409. 10.1007/s11306-011-0325-0

[CR28] Hildebrandt, T. M., Nunes Nesi, A., Araújo, W. L., & Braun, H. P. (2015). Amino Acid Catabolism in Plants. *Molecular Plant*, *8*, 1563–1579. 10.1016/j.molp.2015.09.005 26384576 10.1016/j.molp.2015.09.005

[CR29] Hummel, I., Couée, I., Amrani, E., Martin-Tanguy, A., & Hennion, J., F (2002). Involvement of polyamines in root development at low temperature in the subantarctic cruciferous species Pringlea antiscorbutica. *Journal Of Experimental Botany*, *53*, 1463–1473. 12021294

[CR31] Kong, J. Q. (2015). Phenylalanine ammonia-lyase, a key component used for phenylpropanoids production by metabolic engineering. *Rsc Advances*, *5*, 62587–62603. 10.1039/C5RA08196C

[CR30] Kong, D., Ju, C., Parihar, A., Kim, S., Cho, D., & Kwak, J. M. (2015). Arabidopsis Glutamate Receptor Homolog3.5 Modulates Cytosolic Ca2 + Level to Counteract Effect of Abscisic Acid in Seed Germination. *Plant Physiology*, *167*, 1630–1642. 10.1104/pp.114.251298 25681329 10.1104/pp.114.251298 PMC4378146

[CR32] La Torre, A., Battaglia, V., & Caradonia, F. (2016). An overview of the current plant biostimulant legislations in different European Member States. *Journal Of The Science Of Food And Agriculture*, *96*, 727–734. 10.1002/jsfa.7358 26227817 10.1002/jsfa.7358

[CR33] Lemaire, L., Deleu, C., & Le Deunff, E. (2013). Modulation of ethylene biosynthesis by ACC and AIB reveals a structural and functional relationship between the K15NO3 uptake rate and root absorbing surfaces. *Journal Of Experimental Botany*, *64*, 2725–2737. 10.1093/jxb/ert124 23811694 10.1093/jxb/ert124

[CR36] Li, Z., Zhao, C., Zhao, X., Xia, Y., Sun, X., Xie, W., Ye, Y., Lu, X., & Xu, G. (2018). Deep Annotation of Hydroxycinnamic Acid Amides in Plants Based on Ultra-High-Performance Liquid Chromatography–High-Resolution Mass Spectrometry and Its In Silico Database. *Analytical Chemistry*, *90*, 14321–14330. 10.1021/acs.analchem.8b03654 30453737 10.1021/acs.analchem.8b03654

[CR34] Li, C., Luo, Y., Jin, M., Sun, S., Wang, Z., & Li, Y. (2021). Response of Lignin Metabolism to Light Quality in Wheat Population. *Frontiers In Plant Science*. 12. 10.3389/fpls.2021.729647 PMC8473876 34589105

[CR35] Li, J., Van Gerrewey, T., & Geelen, D. (2022). A Meta-Analysis of Biostimulant Yield Effectiveness in Field Trials. Front. *Plant Science* 13. 10.3389/fpls.2022.836702 PMC9047501 35498677

[CR37] Liliane, T. N., Charles, M. S., Liliane, T. N., & Charles, M. S. (2020). Factors Affecting Yield of Crops, in: Agronomy - Climate Change & Food Security. IntechOpen. 10.5772/intechopen.90672

[CR38] Liu, S., Jiang, J., Ma, Z., Xiao, M., Yang, L., Tian, B., Yu, Y., Bi, C., Fang, A., & Yang, Y. (2022). The Role of Hydroxycinnamic Acid Amide Pathway in Plant Immunity. *Frontiers In Plant Science*, *13*, 922119. 10.3389/fpls.2022.922119 35812905 10.3389/fpls.2022.922119 PMC9257175

[CR39] Macoy, D. M., Kim, W. Y., Lee, S. Y., & Kim, M. G. (2015). Biotic stress related functions of hydroxycinnamic acid amide in plants. *J Plant Biol*, *58*, 156–163. 10.1007/s12374-015-0104-y

[CR40] Macoy, D. M. J., Uddin, S., Ahn, G., Peseth, S., Ryu, G. R., Cha, J. Y., Lee, J. Y., Bae, D., Paek, S. M., Chung, H. J., Mackey, D., Lee, S. Y., Kim, W. Y., & Kim, M. G. (2022). Effect of Hydroxycinnamic Acid Amides, Coumaroyl Tyramine and Coumaroyl Tryptamine on Biotic Stress Response in Arabidopsis. *J Plant Biol*, *65*, 145–155. 10.1007/s12374-021-09341-2

[CR41] Moheb, A., Ibrahim, R. K., Roy, R., & Sarhan, F. (2011). Changes in wheat leaf phenolome in response to cold acclimation. *Phytochemistry*, *72*, 2294–2307. 10.1016/j.phytochem.2011.08.021 21955620 10.1016/j.phytochem.2011.08.021

[CR42] Morreel, K., Dima, O., Kim, H., Lu, F., Niculaes, C., Vanholme, R., Dauwe, R., Goeminne, G., Inzé, D., Messens, E., Ralph, J., & Boerjan, W. (2010). Mass Spectrometry-Based Sequencing of Lignin Oligomers. *Plant Physiology*, *153*, 1464–1478. 10.1104/pp.110.156489 20554692 10.1104/pp.110.156489 PMC2923877

[CR43] Ochoa-Meza, L. C., Quintana-Obregón, E. A., Vargas-Arispuro, I., Falcón-Rodríguez, A. B., Aispuro-Hernández, E., Virgen-Ortiz, J. J., & Martínez-Téllez, M. Á. (2021). Oligosaccharins as Elicitors of Defense Responses in Wheat. *Polymers*, *13*, 3105. 10.3390/polym13183105 34578006 10.3390/polym13183105 PMC8470072

[CR44] Paul, K., Sorrentino, M., Lucini, L., Rouphael, Y., Cardarelli, M., Bonini, P., Reynaud, H., Canaguier, R., Trtílek, M., Panzarová, K., & Colla, G. (2019). Understanding the Biostimulant Action of Vegetal-Derived Protein Hydrolysates by High-Throughput Plant Phenotyping and Metabolomics: A Case Study on Tomato. Front. *Plant Science*. 10. 10.3389/fpls.2019.00047 PMC6376207 30800134

[CR45] Peer, W. A., Bandyopadhyay, A., Blakeslee, J. J., Makam, S. N., Chen, R. J., Masson, P. H., & Murphy, A. S. (2004). Variation in expression and protein localization of the PIN family of auxin efflux facilitator proteins in flavonoid mutants with altered auxin transport in Arabidopsis thaliana. *The Plant Cell*, *16*, 1898–1911. 10.1105/tpc.021501 15208397 10.1105/tpc.021501 PMC514169

[CR46] Pratelli, R., & Pilot, G. (2014). Regulation of amino acid metabolic enzymes and transporters in plants. *Journal Of Experimental Botany*, *65*, 5535–5556. 10.1093/jxb/eru320 25114014 10.1093/jxb/eru320

[CR47] Schütz, L., Gattinger, A., Meier, M., Müller, A., Boller, T., Mäder, P., & Mathimaran, N. (2017). Improving Crop Yield and Nutrient Use Efficiency via Biofertilization-A Global Meta-analysis. Front. *Plant Science*, *8*, 2204. 10.3389/fpls.2017.02204 10.3389/fpls.2017.02204 PMC5770357 29375594

[CR48] Shim, K., & Jeong, Y. (2019). Freshness Evaluation in Chub Mackerel (Scomber japonicus) Using Near-Infrared Spectroscopy Determination of the Cadaverine Content. *Journal Of Food Protection*, *82*, 768–774. 10.4315/0362-028X.JFP-18-529 30978109 10.4315/0362-028X.JFP-18-529

[CR49] Tais, L., Schulz, H., & Böttcher, C. (2021). Comprehensive profiling of semi-polar phytochemicals in whole wheat grains (Triticum aestivum) using liquid chromatography coupled with electrospray ionization quadrupole time-of-flight mass spectrometry. *Metabolomics Off J Metabolomic Soc*, *17*, 18. 10.1007/s11306-020-01761-4 10.1007/s11306-020-01761-4 PMC7840630 33502591

[CR50] Tang, W., & Newton, R. J. (2005). Polyamines promote root elongation and growth by increasing root cell division in regenerated Virginia pine (Pinus virginiana Mill.) plantlets. *Plant Cell Reports*, *24*, 581–589. 10.1007/s00299-005-0021-5 16160835 10.1007/s00299-005-0021-5

[CR51] Tarenghi, E., & Martin-Tanguy, J. (1995). Polyamines, floral induction and floral development of strawberry (Fragaria ananassa Duch). *Plant Growth Regulation*, *17*, 157–165. 10.1007/BF00024176

[CR52] Tchoumtchoua, J., Mathiron, D., Pontarin, N., Gagneul, D., van Bohemen, A. I., Otogo, N., Mesnard, E., Petit, F., Fontaine, E., Molinié, J. X., & Quéro, R., A (2019). Phenolic Profiling of Flax Highlights Contrasting Patterns in Winter and Spring Varieties. *Mol Basel Switz*, *24*, 4303. 10.3390/molecules24234303 10.3390/molecules24234303 PMC6930658 31779076

[CR10] van Butselaar, T., Ackerveken, G. V., & den (2020). Salicylic Acid Steers the Growth–Immunity Tradeoff. *Trends In Plant Science*, *25*, 566–576. 10.1016/j.tplants.2020.02.002 32407696 10.1016/j.tplants.2020.02.002

[CR53] Wan, J., He, M., Hou, Q., Zou, L., Yang, Y., Wei, Y., & Chen, X. (2021). Cell wall associated immunity in plants. *Stress Biol*, *1*, 3. 10.1007/s44154-021-00003-4 37676546 10.1007/s44154-021-00003-4 PMC10429498

[CR54] Yi Chou, E., Schuetz, M., Hoffmann, N., Watanabe, Y., Sibout, R., & Samuels, A. L. (2018). Distribution, mobility, and anchoring of lignin-related oxidative enzymes in Arabidopsis secondary cell walls. *Journal Of Experimental Botany*, *69*, 1849–1859. 10.1093/jxb/ery067 29481639 10.1093/jxb/ery067 PMC6018803

[CR55] Zeiss, D. R., Piater, L. A., & Dubery, I. A. (2021). Hydroxycinnamate Amides: Intriguing Conjugates of Plant Protective Metabolites. *Trends In Plant Science*, *26*, 184–195. 10.1016/j.tplants.2020.09.011 33036915 10.1016/j.tplants.2020.09.011

